# Effectiveness of physical therapy interventions in women with dyspareunia: a systematic review and meta-analysis

**DOI:** 10.1186/s12905-023-02532-8

**Published:** 2023-07-24

**Authors:** Paula Fernández-Pérez, Raquel Leirós-Rodríguez, Mª Pilar Marqués-Sánchez, María Cristina Martínez-Fernández, Fernanda Oliveira de Carvalho, Leonardo Y. S. Maciel

**Affiliations:** 1 Nursing and Physical Therapy Department, University of Leon, Astorga Ave, 24401 Ponferrada, Spain; 2 SALBIS Research Group, Nursing and Physical Therapy Department, University of Leon, Astorga Ave, 24401 Ponferrada, Spain; 3 Brazilian Hospital Services Company (Ebserh) R. Cláudio Batista - Palestina, Aracaju, 49060-676 Brazil; 4 Physical Therapy Department of Lagarto, Universidade Federal de Sergipe, Gov. Marcelo Déda Ave, São José, Lagarto 49400-000 Brazil; 5 Faculty of Sport, Research Centre in Physical Activity, Health and Leisure (CIAFEL), University of Porto, R. Dr. Plácido da Costa 91, 4200-450 Porto, Portugal

**Keywords:** Dyspareunia, Physiological sexual dysfunctions, Physiological sexual disorders, Pelvic floor, Physical therapy modalities, Rehabilitation, Exercise therapy, Manual therapy

## Abstract

**Background:**

Dyspareunia is defined as the occurrence of pain during or after sexual intercourse, which directly affects physical, sexual, and mental health. This condition can lead to depression, anxiety, and low self-esteem in women who experience it.

**Objectives:**

The aim of this research was to evaluate the effectiveness of physical therapy interventions for the treatment of female dyspareunia.

**Design:**

A systematic review and meta-analysis was conducted.

**Method:**

Search of publications was conducted in Scopus, Medline, Pubmed, Cinahl and Web of Science. Treatment effects were defined as standardized mean difference and their 95% confidence intervals. Statistical heterogeneity was assessed using Crohan's Q test and quantified using the I^2^ index.

**Results:**

Of the 19 articles selected, six applied multimodal physiotherapy treatments; five, electrotherapy; three, Thiele's massage; two, interdisciplinary interventions or pelvic floor muscle training; and one, extracorporeal shockwave therapy. The meta-analysis showed significant results for the variables pain and quality of life with the interventions based on electrotherapy and electrotherapy combined with pelvic floor muscle training. These interventions did not show significant results for the improvement of sexual function.

**Conclusions:**

Physiotherapy techniques are effective and procedures have been identified with reliable results in improving pain and quality of life in patients with dyspareunia. One of the most important aspects is the strengthening of the perineal musculature and the application of Transcutaneous Electrical Nerve Stimulation. Furthermore, manual trigger point release therapy and Thiele massage, optimize and guarantee the reduction of pain intensity.

**Prospero registration:**

CRD42021236155.

## Background

Female sexual dysfunction is known as the disorder experienced by a woman when changes occur in her usual sexual behavior [1]. It is estimated that between 16 and 40% of women suffer from some form of sexual dysfunction, and this percentage increases with age [2]. Among the painful disorders, dyspareunia stands out. It involves the onset of pain during or after intercourse, directly affecting physical health, as well as sexual and mental well-being. Consequently, it can lead to depression, anxiety, and low self-esteem in women who experience it [3]. The prevalence of distressing sexual problems in women peaked at 14.8% in middle-aged women compared to younger women (aged 18–44 years: 10.8%) or older women (aged ≥ 65 years: 8.9%) [4]. Dyspareunia can be classified as superficial when it affects the vulva and vaginal entrance, or deep when the painful area is the cervix, bladder, and/or lower pelvis. Another classification divides it into primary, associated with pain at the beginning of sexual life, and secondary, when it appears at a later time [5].

Specifying the etiology of dyspareunia can be challenging as it encompasses structural, inflammatory, infectious, traumatic, hormonal, and psychosocial conditions [5, 6]. These conditions can act as both risk factors and consequences, creating a cycle that is influenced by emotional intimacy, sexual stimuli, arousal, and physical and emotional satisfaction in a non-linear manner. The disruption of this cycle predisposes individuals to experience sexual pain [7].

The treatment of sexual dysfunctions does not guarantee complete resolution, but it does help reduce their impact on patients' quality of life [8]. One of the most recommended approaches is multidisciplinary treatment, which addresses physical, emotional, and behavioral aspects. This approach involves a multidisciplinary team comprising gynecologists, physiotherapists, sex therapists, and psychologists and/or psychiatrists. Among them, physiotherapy for sexual dysfunctions has the ability to enhance sexual health through individualized interventions for each patient. These interventions include education on healthy habits, promotion of active lifestyles, improvement of self-image and body appreciation, and enhancement of the biomechanics and physiology of pelvic-perineal structures [9].

However, to date, there has been no detailed definition of which techniques are most suitable for treating dyspareunia, including their optimal application parameters or dosage. Therefore, this systematic review and meta-analysis were considered necessary to evaluate the effectiveness of physical therapy interventions for the treatment of female dyspareunia.

## Methods

### Eligibility criteria, information sources and search strategy

This systematic review and meta-analysis were prospectively registered on PROSPERO (ID: CRD42021236155) and followed the reporting guidelines of the Preferred Reporting Items for Systematic Reviews and Meta-analyses (PRISMA) in Exercise, Rehabilitation, Sport Medicine, and Sports Science, as well as the recommendations from the Cochrane Collaboration [10, 11]. The PICO question was formulated as follows: P – population: women with dyspareunia; I – intervention: physical therapy techniques; C – control: pharmacological treatment, psycho-behavioral interventions, or non-intervention; O – outcome: intensity of perceived pain during sex and strength and elasticity of the perineal muscles; S – study designs: experimental studies (quasi-experimental and clinical trials).

A systematic search of publications was conducted in May 2023 using the following databases: Scopus, Medline, PubMed, CINAHL, and Web of Science. The search strategy involved various combinations of Medical Subject Headings (MeSH) terms: *Dyspareunia, Sexual dysfunctions, Pelvic floor, Physical therapy modalities, *

#### Rehabilitation, exercise and manual therapy

The search strategy, aligned with the focused PICOS question, is presented in Table 1.

**Table 1:** Search strategy according to the focused question (PICO)

Database	Search equation
PubMed	"Pelvic floor"[Mesh] AND "Physiological sexual dysfunctions"[Mesh] AND "Physical therapy[Title/Abstract]" AND "Women[Title/Abstract]" “Exercise therapy”[Mesh] AND "Physiological sexual dysfunctions"[Mesh] AND "Women[Title/Abstract]” “Dyspareunia”[Mesh] AND “Rehabilitation”[Mesh] “Dyspareunia”[Mesh] AND “Physical therapy modalities”[Mesh] “Dyspareunia”[Mesh] AND “Manual therapy”[Mesh]
ScienceDirect	Title, abstract, keywords: ("Pelvic floor") AND ("Physiological sexual dysfunctions)" AND ("Physical therapy") AND ("Women") Title, abstract, keywords: ("Exercise therapy") AND ("Physiological sexual dysfunctions") AND ("Women") Title, abstract, keywords: ("Dyspareunia") AND ("Physical therapy modalities”) Title, abstract, keywords: ("Dyspareunia") AND ("Rehabilitation") Title, abstract, keywords: ("Dyspareunia") AND (“Manual therapy")
Cinahl	MH "Pelvic floor" AND MH "Physiological sexual dysfunctions" AND AB "Physical therapy" AND AB "Women" MH “Exercise therapy” AND MH "Physiological sexual dysfunctions" AND AB "Women” MH “Dyspareunia” AND MH “Rehabilitation” MH “Dyspareunia” AND MH “Physical therapy modalities” MH “Dyspareunia” AND MH “Manual therapy”[Mesh]
Web of Science	((TS = (Physical therapy) AND TS = (Physiological sexual dysfunctions)) AND TS = (Women)) ((TS = (Exercise therapy) AND TS = (Physiological sexual dysfunctions)) AND TS = (Women)) ((TS = (Dyspareunia) AND TS = (Physical therapy modalities)) ((TS = (Dyspareunia) AND TS = (Rehabilitation)) ((TS = (Dyspareunia) AND TS = (Manual therapy)) ((TS = "Exercise therapy") AND (TS = "Dyspareunia"))
Scopus	TITLE-ABS-KEY ( "Pelvic floor") AND TITLE-ABS-KEY ( "Physiological sexual dysfunctions") AND TITLE-ABS-KEY ( "Physical therapy") AND TITLE-ABS-KEY ( "Women") TITLE-ABS-KEY ( “Exercise therapy”) AND TITLE-ABS-KEY ( "Physiological sexual dysfunctions") AND TITLE-ABS-KEY ( "Women”) TITLE-ABS-KEY ( “Dyspareunia”) AND TITLE-ABS-KEY ( “Rehabilitation”) TITLE-ABS-KEY ( “Dyspareunia”) AND TITLE-ABS-KEY ( “Physical therapy modalities”) TITLE-ABS-KEY ( “Dyspareunia”) AND TITLE-ABS-KEY ( “Manual therapy”)

### Study selection

After removing duplicates, two reviewers (X. X.-X. and X.X.-X.) independently screened the articles for eligibility. In cases of disagreement, a third reviewer (X. X.-X.) made the final decision on whether to include the study or not. The inclusion criteria established that: (a) the intervention should involve at least one physical therapy technique or treatment method; (b) the sample should consist exclusively of women; and (c) the sample should include patients with dyspareunia. On the other hand, studies with non-experimental methodologies (such as reviews, meta-analyses, editorials, etc.) and those without full-text availability were excluded from this review.

After screening the data, extracting, and obtaining the titles and abstracts based on the inclusion criteria, the selected abstracts were obtained in full text. Titles and abstracts lacking sufficient information regarding the inclusion criteria were also obtained as full texts. Full-text articles were selected by the two reviewers if they met the inclusion criteria, using a data extraction form.

### Data synthesis

The two reviewers independently extracted data from the included studies using a customized data extraction table in Microsoft Excel. In case of disagreement, both reviewers engaged in discussions and debates until an agreement was reached.

### Data extraction

The data extracted from the included articles for further analysis included demographic information (title, authors, journal, and year), characteristics of the sample (age, inclusion and exclusion criteria, and number of participants), study-specific parameters (study type, duration of the intervention, physical therapy techniques applied), and results obtained (variables analyzed, instruments used, and follow-up time). When possible, the results were categorized based on the type of intervention applied. Tables were used to describe both the characteristics of the studies and the extracted data.

### Assessment of risk of bias

The ROBINS-I tool was used to assess the risk of bias in non-randomized studies [12], while the RoB tool was used to assess the risk of bias in randomized studies [13]. Additionally, the Grades of Recommendations Assessment, Development, and Evaluation (GRADE) approach was employed to assess the quality of the evidence when conducting meta-analysis [14].

### Statistical analysis

The treatment effects were defined as standardized mean differences (SMD) along with their corresponding 95% confidence intervals (CI). The mean and standard deviation (SD) were obtained for each study group, and the effect size was calculated based on the outcome of interest. A random-effects model, assuming heterogeneity across studies, was developed using the DerSimonian and Laird method. The magnitude of the effect size of the intervention was assessed using Cohen's method, where an SMD between 0.2 and 0.5 indicates a small effect, between 0.5 and 0.8 indicates a moderate effect, and an SMD greater than 0.8 indicates a large effect. Forest plots were used to visually represent the effect sizes and their corresponding 95% confidence intervals (CI). Statistical significance was set at *p* < 0.05.

The model described by Higgins and Green was utilized to calculate the mean and SD between pre- and post-treatment data for all studies included in the meta-analysis [15]. Statistical heterogeneity was assessed using Cochran's Q test and quantified using the I^2^ index [16]. A subgroup analysis was conducted based on the type of treatment analyzed, specifically electrotherapy or a combination of electrotherapy and kinesitherapy. All analyses were performed using Review Manager 5.3 (The Cochrane Collaboration, 2014).

## Results

### Study selection and characteristics

**Fig. 1:**
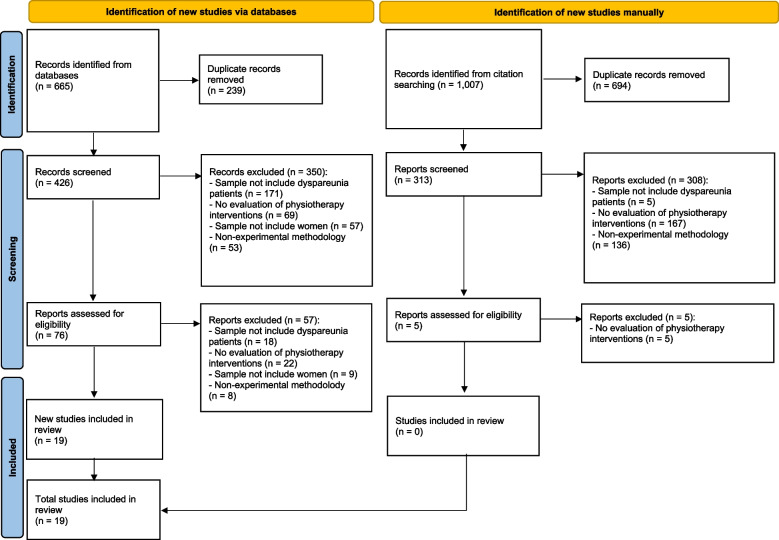
Preferred Reporting Items for Systematic Reviews and Meta-Analyses (PRISMA) flow diagram

The systematic search of publications resulted in a total of 1,672 results. After removing duplicate results, the abstract titles of 713 publications were analyzed. From this initial analysis, 81 results were selected for full-text analysis. The agreement between reviewers 1 and 2, as measured by the Kappa score, was 0.9, indicating a very high level of agreement. Finally, based on the defined inclusion and exclusion criteria, 19 articles were considered eligible and included in the review (see Fig. 1).

Of the 19 articles, six applied multimodal physiotherapy treatments [17–22]; five studies utilized electrotherapy [23–27] (with two of them combined with drugs [24, 25] and two with pelvic floor muscle training [26, 27]); three studies used Thiele's massage [28–30] (with one of them including an educational session [30]); and two studies implemented an interdisciplinary intervention [31, 32] or pelvic floor muscle training [33, 34]. Lastly, one study exclusively applied extracorporeal shockwave therapy [35].

The methodological characteristics of the studies are presented in Table 2, while a summary of the findings from each study can be found in Table 3.

**Table 2:** Methodological characteristics of the studies analyzed

**Authors**	**Design**	**Sample size**	**Intervention**	**Time of intervention (Frequency of sessions)**
Alshiek et al. (2017) [26]	QES	94	Abdominal and vaginal electromyography. Vaginal electrogalvanic stimulation and biofeedback	8 – 14 weeks (1 session every two weeks)
Brotto et al. (2015) [32]	QES	132	Patient education. Biofeedback. Use of vaginal accommodators. Psychological treatment. Pelvic floor relaxation. Active home pelvic floor muscle training	10–12 weeks (*not described*)
Cyr et al. (2020) [19]	QES	31	Patient education. Manual therapy. Active pelvic floor muscle training assisted with biofeedback Active pelvic floor muscle training at home	12 weeks (1 session each week)
Cyr et al. (2021) [22]				
Cyr et al. (2022) [21]				
Da Silva et al. (2016) [29]	QES	18	Thiele massage	4 weeks (1 session each week)
Del Forno et al. (2020) [28]	QES	10	Ultrasound biofeedback. Thiele massage	11 weeks (1 session weekly in the 1^st^, 3^rd^, 5^th^, 8 ^th^ and 11^th^ weeks)
Del Forno et al. (2021) [30]	RCT	34	Experimental group: Education and Thiele massage Control group:	
Fernández-Cuadros et al. (2020) [27]	QES	37	Pelvic floor muscle training assisted with manometric biofeedback. Radiofrequency at 448 kHz (bipolar capacitive in abdomen and perineum and intravaginal and lumbo-sacral resistive)	4 weeks (2 sessions each week)
Franco et al. (2021) [34]	RCT	77	Experimental group: Pelvic floor muscle training (supervised and at home) Control group: no treatment	12 weeks (2 sessions each week)
Ghaderi et al. (2019) [17]	RCT	64	Experimental group: intravaginal electrotherapy (transcutaneous nerve electrostimulation at 110 Hz, 80 ms pulses and maximum tolerated intensity). Manual therapy and pelvic floor muscle training Control group: no treatment	12 weeks (1 session each week)
Hurt et al. (2021) [35]	RCT	62	Experimental group: Extracorporeal shock wave therapy Control group: Sham extracorporeal shock wave therapy	4 weeks (1 session each week)
Kolberg et al. (2015) [33]	RCT	145	Experimental group: Pelvic floor muscle training Control group: home pelvic floor muscle training (3 series of 8–12 maximum contractions)	16 weeks (Experimental group: 1 session each week)
**Authors**	**Design**	**Sample size**	**Intervention**	**Time of intervention** **(Frequency of sessions)**
Mira et al. (2015) [23]	RCT	22	Transcutaneous nerve electrostimulation: Experimental group: at 8 Hz and 250 ms pulses Control group: self-applied at 85 Hz and 75 ms pulses	8 weeks (not described)
Mira et al. (2020) [24]	RCT	42	Experimental group: Hormone pelvic floor muscle training and electrotherapy Control group: Hormone therapy	8 weeks (not described)
Morin et al. (2021) [20]	RS	212	Group 1: Education. Manual therapy. Pelvic floor muscle training. Stretching with dilators Group 2: overnight topical lidocaine	10 weeks (1 session each week)
Murina et al. (2018) [25]	RCT	101	Transcutaneous nervous electrostimulation at 100 Hz, 50 and 100 ms pulses during 15 min: Experimental group: combined with Diazepam Control group: combined with placebo	6 weeks (3 sessions each week)
Schvertzman et al. (2019) [18]	RS	42	Group 1: thermotherapy, myofascial release, active kinesitherapy and electromyography Group 2: thermotherapy, myofascial release of the abdominal diaphragm, piriformis and psoas iliacus	5 weeks (*not described*)
Yong et al. (2018) [31]	CS	278	Psychological treatment. Gynecological therapies; physiotherapeutic treatment of factors affecting sexual function, relaxation techniques and diaphragmatic breathing Pain education, pelvi-perineal anatomy and sexual function	1 year (*not described*)

*RS* Randomized study, *CS* Controlled study, *RCT* Randomized controlled trial, *QES* Quasi-Experimental study

**Table 3:** Characteristics and results of the studies analyzed

Authors	Objectives	Inclusion criteria	Exclusion criteria	Improvements identified
Alshiek et al. (2017) [26]	To evaluate a Physical Therapy program that included behavior modification, biofeedback and vaginal electrogalvanic stimulation	Women with urinary urgency or increased frequency, urinary and/or fecal incontinence; defecatory dysfunction; pelvic pain; perineal muscle dysfunction and dyspareunia	Have an implanted electrical device	Reduction of pain, nocturia, urine leakage and intestinal symptoms
Brotto et al. (2015) [32]	To evaluate a hospital-based intervention that integrated psychological skills training, physical therapy, and medical management on sexual function	Women with diagnosis of vulvodynia, in reproductive age, with dyspareunia of at least 6 months of evolution and ability to participate in group sessions	Being in menopausal state. Unprovoked chronic discomfort, dyspareunia due to other etiology. Presence of language barrier. Non-participatory patients	Reduction of distress, pain intensity and dyspareunia symptoms Increased sexual functioning
Cyr et al. (2020) [19]	To examine the effects of multimodal physical therapy in gynecologic cancer survivors	Women over 13 years of age and between 6 and 13 weeks of gestation. Pregnancy follow-up in one of the eight study hospitals included	Presence of pelvic pain not associated with cancer, urinary or vaginal tract infection, chronic constipation, grade III genitourinary prolapse, psychological condition and/or other types of cancer. History of dyspareunia prior to cancer, vulvar, vaginal or pelvic surgery unrelated to gynecologic cancer, physical therapy treatment within the last year. Changes in hormonal therapy in the last 6 months. Patient's refusal	Reduction of pain intensity, urinary and vaginal symptoms Increased sexual function and quality of life and frequency of penetrative intercourse
Cyr et al. (2021) [22]				Reduction of sexual distress, body image concerns, pain anxiety, pain catastrophizing and depressive symptoms Increased painful intercourse self-efficacy
Cyr et al. (2022) [21]				Reduction in levator hiatal area and anterior–posterior diameter on maximal contraction (improved contractility) Increased anorectal angle and the levator hiatal dimension at rest (reduction in muscle tone), bladder neck more cranially and ventrally on maximal contraction
Da Silva et al. (2016) [29]	To evaluate the long-term effectiveness of Thiele massage in pelvic tenderness dyspareunia	Women in reproductive age, sexually active and diagnosed with dyspareunia due to pain on palpation of the perineal muscles	Presence of cognitive disorders; diabetes mellitus; neuropathies; vasculopathy and/or pelvic organ prolapse. Use of antidepressants	Reduction of pain intensity Increased quality of pain, sexual function (not in the chronic pain group)
Del Forno et al. (2020) [28]	To evaluate the effects of physical therapy in women with deep infiltrating endometriosis and associated dyspareunia	Women aged 18 to 45 years with a diagnosis of deep infiltrating endometriosis and associated dyspareunia	History of genital disease, pelvic organ prolapse, previous surgery; pelvi-perineal anomalies and/or other causes of chronic pelvic pain	Reduction of pain intensity Increased activity of the levator ani
Del Forno et al. (2021) [30]	To evaluate the effect of pelvic floor Physiotherapy on the levator hiatal area during Valsalva maneuver in women with deep infiltrating endometriosis suffering from superficial dyspareunia	Women aged 18 to 45 years with clinical and ultrasound diagnosis of deep infiltrating endometriosis according to the criteria of the International Deep Endometriosis Analysis Group, and associated superficial dyspareunia	Previous or current genital malignancy, pelvic organ prolapse, previous surgery for deep infiltrating endometriosis, current or previous pregnancy, congenital or acquired abnormalities of the pelvis or pelvic floor, history of sexual abuse, current genitourinary infection and presence of other causes of chronic pelvic pain	Reduction of pain intensity Increased levator hiatal area on maximum Valsalva maneuver (better pelvic floor muscle relaxation)
Fernández-Cuadros et al. (2020) [27]	To demonstrate whether a multimodal rehabilitation protocol is effective in patients with chronic pelvic pain dyspareunia	Women over 18 years of age and diagnosis of chronic pelvic pain and/or dyspareunia of more than 6 months of evolution. Patients referred to Rehabilitation from Gynecology, Psychiatry, Psychology, Urology or Primary Care	Presence of difficulty in comprehension and/or collaboration, neurological conditions affecting muscle contraction, contraindications for radiofrequency and/or thermotherapy. Failure to perform any of the assessment tests	Decrease in pain intensity Increase of perineal strength
Franco et al. (2021) [34]	To evaluate the effect of pelvic floor muscle training on sexual function in postmenopausal women	Women with maximum of 5 years of postmenopause, with intercourse with penile penetration into the vagina in the last month, ability to perform a voluntary pelvic floor muscle contraction (≥ grade 1 according to the modified Oxford scale), to be in a stable relationship with the partner for at least 4 months	Uso de hormonal replacement therapy. Presencia de intolerance (pain or any other discomfort) during the pelvic floor muscle function examination. Refuse to answer the self-report questionnaires Presence of pelvic organd prolapse of more than grade 1 Diabetes mellitus inestable, thyroid disease, hyperprolactinemia, neuropathy, and vasculopathy according to their report	No change in sexual function
Ghaderi et al. (2019) [17]	To evaluate the effects of a rehabilitation intervention	Presence of pain in the genital area before, during or after vaginal intercourse of an intensity greater than 8 on the Visual Analog Scale	History of pathophysiological conditions (infections, tumors, psychiatric diseases, vaginismus, vestibulodynia, vulvar dermatological conditions, painful bladder syndrome or cystitis, endometriosis, pregnancy, pelvic surgery). Simultaneous performance of another treatment for dyspareunia	Reduction of pain intensity. Increased perineal strength and endurance and sexual function
Hurt et al. (2021) [35]	To determine whether extracorporeal shock waves therapy is effective for treating dyspareunia in women	Painful penile-vaginal penetration without pelvic organic reasons primarily connected to pain, a score of > 0 on the Marinoff Dyspareunia Scale and on a visual analog scale. Women aged between 20 and 75 years old. Duration of dyspareunia > 3 months during the past 6 months. Reduction of pain was unobtainable by other therapeutic approaches	Acute pelvic inflammation during the past 6 months, oncological disease within the past 5 years, clinically significant haematologic disease, myocardial infarction or cardiac arrhythmia within the past 6 months, any serious metabolic disorder and affection in an intended application area	Reduction of pain intensity and dyspareunia
Kolberg et al. (2015) [33]	To evaluate the effectiveness of perineal training in primiparous women with significant levator ani defects	Primiparous women with single vaginal delivery after more than 32 weeks of gestation and with understanding of Scandinavian languages	History of cesarean delivery, perineal tears grade IIIb or higher. Presence of severe illness of the mother and/or neonate	Increased strength and endurance of the levator ani
Mira et al. (2015) [23]	To evaluate the effectiveness of transcuataneous nerve electrostimulation for the relief of dyspareunia with deep endometriosis	Women in menacme, aged between 18 and 50 years, with a diagnosis of deep endometriosis in the cul-de-sac and bowel loop and pelvic pain and/or dyspareunia	Women with decreased skin sensitivity and/or implanted with pacemakers	Reduction of pain Increased quality of life Better results in the group that received high frequency
Mira et al. (2020) [24]	To evaluate the effectiveness of adjunctive treatment to hormonal therapy in women with deep endometriosis	Women in menacme, diagnosed with deep endometriosis by transvaginal ultrasound or MRI, under continuous hormone therapy for at least three months and presence of symptoms of chronic pelvic pain and/or dyspareunia	Presence of pregnancy, pacemaker, decreased skin sensitivity, allergy to conductive gel or electrodes, epilepsy, cardiac arrhythmia, osteosynthesis in the treatment region, cancer, pelvic inflammatory disease and/or cognitive developmental impairment	Reduction of pain intensity, dyspareunia, Increased sexual function and quality of life
Morin et al. (2021) [20]	To determine the efficacy of physical therapy in women with provoked vestibulodynia compared with overnight topical lidocaine	Nulliparous women, aged 18 to 45 years, pain during sexual intercourse for > 6 months with an average intensity of more than 5 of 10 on a numeric rating scale. Diagnosis of provoked vestibulodynia confirmed by gynecologists according to current	Presence of other urogynecologic and vulvar pain conditions. Previously received physical therapy or overnight lidocaine, and any coexisting significant medical conditions that were likely to interfere with the study procedures	Reduction of sexual distress, pain intensity and quality Increased sexual function
Murina et al. (2018) [25]	To evaluate the effectiveness of transcuataneous electrostimulation and vaginal Diazepam in the treatment of vulvodynia	Diagnosis of vulvodynia, vulvar pain or dyspareunia in women over 18 years of age and diagnosis of moderate or severe pelvic disease and/or hypertonic dysfunction	Presence of pregnancy and/or contraindications for the consumption of diazepam or any benzodiazepine	Reduction of dyspareunia and resting tone Increased sensory threshold
Schvertzman et al. (2019) [18]	To evaluate the efficacy of a treatment for climacteric women with complaints of dyspareunia	Women between 40 and 60 years of age, sexually active (at least one sexual intercourse in the previous month), with complaints of dyspareunia of at least 6 months duration (of at least a 3 on the Visual Analog Scale) and diagnosis of early perimenopause or menopause	Diagnosis of vaginismus and/or vulvar vestibulitis syndrome. Presence of neurological and/or psychiatric disorders, difficulty in comprehension, pelvic organ prolapse, vaginal bleeding, vaginal atrophy and/or deep endometriosis. History of previous perineal surgery without evidence of pelvic diaphragm contraction and/or physical therapy treatments in the last six months	Reduction of pain intensity Increased sexual function, quality of life and pelvic musculature characteristics
Yong et al. (2018) [31]	To analyze the severity of deep dyspareunia one year in an interdisciplinary center and to identify predictive factors	Women under 50 years of age and sexually active	Diagnosis of menopause	Reduced severity of moderate and mild prodromal dyspareunia. Increased quality of life Depression was identified as having a direct influence on the persistence of dyspareunia

Regarding the experimental designs of the analyzed studies, eight of them were randomized controlled trials [17, 23–25, 30, 33–35], while the remaining studies were quasi-experimental. Among the quasi-experimental studies, eight had a single experimental group [19, 21, 22, 26–28, 31, 32], and three had two experimental groups [18, 20, 29] (with only two of them having random assignment of participants [18, 20]).

### Interventions applied

In the study by Brotto et al. [32], the multidisciplinary intervention included two educational seminars, psychological treatment, and physiotherapy, which involved biofeedback and guidance on pelvic floor relaxation, the use of dilators, and home exercises. In other studies, the intervention included sessions with the gynecologist, physiotherapist, and psychologist. The physiotherapy sessions focused on relaxation techniques, diaphragmatic breathing, addressing central and local factors affecting sexual function (such as sexual interest, desire, arousal), and providing pain education [31]. Both investigations reported significant reductions in pain intensity [31, 32], severity of dyspareunia [32] and sexual distress [32]. They also found statistically significant improvements in sexual quality of life [31] and sexual functionality, including sexual desire, arousal, lubrication, orgasms, sexual satisfaction, and pain reduction [32]. However, no changes were observed on the Dyadic Adjustment Scale [32]. These improvements were still present up to six months after the intervention in the variables of pain, distress, sexual desire, and satisfaction [32].

The investigations that applied multimodal interventions included various techniques such as Transcutaneous Electrical Nerve Stimulation (TENS) (by intravaginal probe at 110 Hz, pulses of 80 ms duration and maximum tolerable intensity) [17], infrared therapy [18], myofascial release of trigger points [17–20], of the abdominal diaphragm, piriformis and psoas-iliac [18, 20], intravaginal massage [17, 19, 20, 22], perineal stretching [19, 20, 22], abdominal training [18], pelvic floor muscle training (contraction and relaxation exercises) [17, 18] and pelvic floor muscle training by biofeedback [18–22] (with electromyography in three of the cases [18, 21, 22]). In addition, in some cases, the researchers provided educational materials such as information leaflets and videos on pelvic floor muscle training [17, 19–22]. Four of these interventions [19–22] also included education for participants on the pathophysiology and management of their dysfunction, as well as the use of lubricants and moisturizers. Additionally, one study recommended self-perineal stretching exercises using a dilator [20]. These multimodal interventions resulted in significant improvements in pain [17–20, 22], sexual function [17, 18, 20] (especially in desire, satisfaction and pain in those participants who received abdomino-pelvic floor muscle training [18]), sexual distress [22], quality of life [18, 19] (if abdomino-pelvic floor muscle training was included [18]), severity of dyspareunia (if abdomino-pelvic floor muscle training was included [18]), perineal function and contractility [17, 18, 21] (more so if the intervention included pelvic floor muscle training [18]) catastrophism, and anxiety [22]. These improvements were still present three months after the end of the intervention [17]. Additionally, Schvartzman et al. [18] using electromyography of the abdominal and perineal musculature, identified a significant increase in sustained contraction time and a reduction in resting activity. However, the intervention by Cyr et al. [19], although it improved sexual functionality and the frequency of penetrative intercourse, did not show significant changes.

Thiele's massage involves a transvaginal massage using longitudinal slides along each muscle with a pressure that is tolerable for the patient. One of its advantages is its ease of learning, allowing the patient and her partner to perform it at home, and it has no contraindications [36]. Research studies utilizing this method have shown significant improvements in pain [28–30] (in one study, only in the intensity of superficial dyspareunia but not deep dyspareunia [30]), sexual desire and lubrication [26] as well as levator ani contractility [28, 30]. These improvements were still present up to six months after the completion of the study [29]. Participants reported being "very satisfied" with the intervention they received [28, 30]. However, no significant changes in anxiety and depression levels were observed [29].

Two of the studies that applied electrotherapy combined the use of Transcutaneous Electrical Nerve Stimulation (TENS) with other treatments [24, 25]. In Murina et al.'s study [25] TENS was combined with vaginal administration of Diazepam, while Mira et al.'s study [24] combined TENS with hormonal therapy. In Murina et al.'s study [25], TENS was administered in two programs. The first program had a frequency of 100 Hz and a pulse duration of 50 ms, while the second program had a frequency of 5 Hz and pulse duration of 100 ms. In Mira et al.'s study [24], TENS was self-administered at home twice a day for 20 min, with a frequency of 85 Hz and a pulse duration of 75 ms. The intensity of TENS was set at 10, 20, or 30 mA based on the patient's preference, up to the maximum non-painful sensory threshold. Both studies reported significant improvements in pain intensity [24, 25] (with the application of TENS without Diazepam there were improvements, although not significant [25], and the combined application with hormonal therapy was significantly superior to hormonal therapy alone [24]), in the severity of dyspareunia [24, 25] (the combined application with hormonal therapy was significantly superior to hormonal therapy alone [24]), in the number of days with pain [24], in the tone of the levator ani, in the number of days with pain [25] (with the application of TENS alone without combination with Diazepam there were non-significant improvements [25]), satisfaction and lubrication related to sexual functionality (satisfaction improved significantly more with the combined application of TENS and hormone therapy than with the latter alone [24]) and quality of life (the combined application with hormone therapy was significantly superior to hormone therapy alone [24]). Sensory threshold, indicative of perineal peripheral nerve integrity, improved with TENS alone and in combination with Diazepam, with greater improvement observed in the TENS-alone group [25] Muscle strength and relaxation capacity of the perineal muscles also improved after TENS and Diazepam treatment, although not statistically significantly [25]. These changes were sustained even two months after the completion of treatment sessions [25].

In one study, the effectiveness of TENS was evaluated based on whether it was applied in the office or at home [23]. In-office TENS was applied at 8 Hz, 260 ms, and intensity at the maximum non-painful threshold. The participants who applied TENS at home used a frequency of 86 Hz, pulse duration of 75 ms, and intensity of 10, 20, or 30 mA. TENS was applied twice a day for 20 min, with a 12-h interval between the two sessions. The electrodes were placed in the sacral region (S3-S4) using two routes with two electrodes each. The study found that all participants experienced a significant reduction in pain, severity of dyspareunia, dyschezia, and improvement in quality of life. Although dysmenorrhea and dysuria improved, the changes were not statistically significant. There were no significant differences between the in-office and at-home TENS groups, but the group that received a higher frequency achieved better results.

Two other studies combined pelvic floor muscle training with electrotherapy interventions [26, 27] (specifically vaginal electrogalvanic stimulation [26] and radiofrequency [27]). Alshiek et al.’s [27] intervention included pelvic floor muscle training with biofeedback, vaginal electrogalvanic stimulation at a variable frequency and tolerable amplitude, and patient education. Fernández-Cuadros et al.’s [27] intervention included pelvic floor muscle exercises assisted with manometric biofeedback of the tonic type (three seconds of work and six seconds of rest) and phasic type (five rapid contractions followed by ten seconds of rest), bipolar capacitive radiofrequency at the suprapubic and perineovaginal level for five minutes (448 kHz) with two electrodes (one on the lower abdomen and the other on the perineum) and vaginal and lumbo-sacral resistive for ten minutes with two electrodes (one active intracavitary vaginal electrode and one passive electrode on the lumbo-sacral region) using INDIBA® (INDIBA, Barcelona, Spain). Both interventions resulted in significant improvements in pain intensity [26, 27], perineal strength [27], intestinal symptoms [26], and urinary symptoms (number of leaks per day, daily episodes of urgency without leaks and with leaks) [26]. However, the study using vaginal electrogalvanic stimulation did not show improvements in intestinal or sexual function [26], while the study using radiofrequency treatment did not improve perineal contractility assessed by manometry [27].

Two studies specifically focused on evaluating the effects of pelvic floor muscle training in different populations. The first study targeted primiparous women with postpartum vaginal dyspareunia [33] while the second study focused on postmenopausal women [34]. In the study involving primiparous women, the participants were divided into two groups: one group received training sessions led by a physiotherapist and a home exercise guide, while the control group only received the home exercise guide [33]. In the other study, the experimental group underwent pelvic floor muscle training both in clinical sessions with the physiotherapist and at home [34]. The training programs in both studies resulted in improvements in perineal muscle function [34] and perineal strength and endurance (more so in the intervention group, although not significantly) [33]. However, there was no significant difference in resting vaginal pressure [33] or sexual function [34]. Additionally, the authors compared women with and without levator ani dysfunction and found that regardless of the presence of muscle defects, pelvic floor muscle strength and endurance significantly improved [33].

A study evaluated the efficacy of extracorporeal shockwave therapy [35]. In this study, the intervention was compared to a placebo group where a pad was placed to block the transmission of impulses between the device and the skin surface. Hurt et al. applied extracorporeal shockwaves on a weekly basis, delivering 4000 pulses per week for 4 consecutive weeks. The energy flux density, frequency, focus zone, therapeutic efficiency, and stand-off parameters were set according to specific values. The shockwave transducer position was changed after every 500 pulses, and eight areas covering the entire vulva and perineum were treated.

The results of the study showed significant changes in penile-vaginal sexual intercourse (assessed using the Marinoff Dyspareunia Scale) and pain intensity (evaluated using the Visual Analogue Scale) following the intervention and up to three months later. However, no significant modifications in these outcomes were observed in the placebo group.

### Meta-analysis results

Despite the methodological variability of the studies, a meta-analysis was conducted to evaluate pain, sexual function, and quality of life in studies comparing physical therapy (including electrotherapy and subgroups with training) with a control group. Due to variations in outcome measures across studies, not all of them were included in the meta-analysis.

**Fig. 2:**
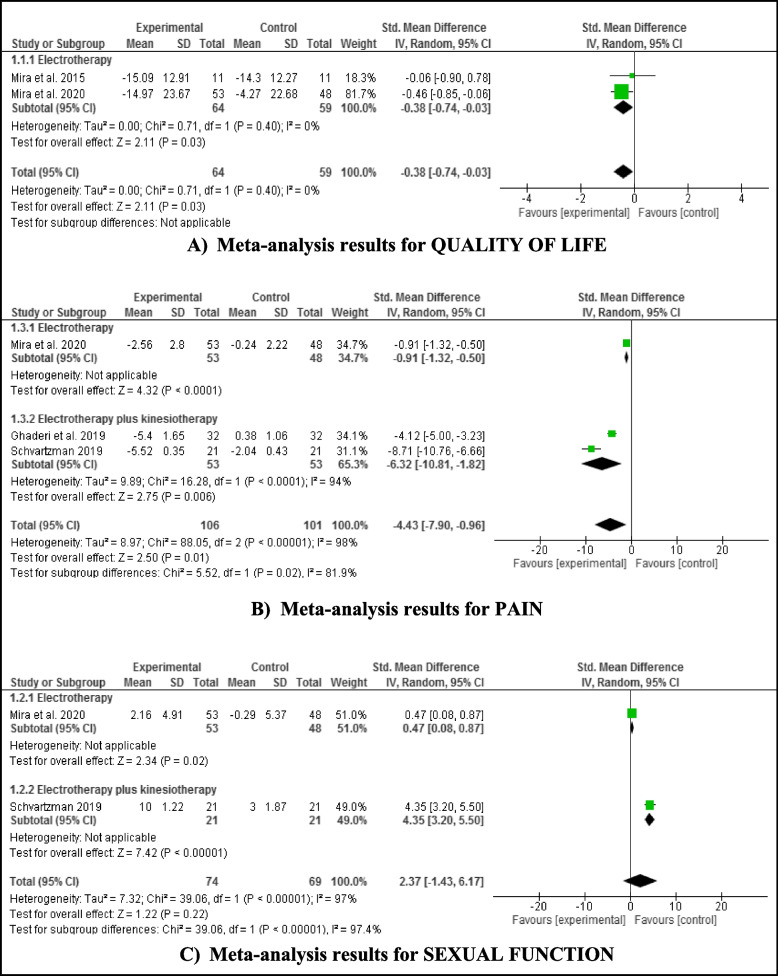
Meta-analysis results for physiotherapy interventions vs. Control group

When analyzing quality of life, the SMD was -0.38 (95% CI: -0.74 to -0.03), indicating that the groups treated with electrotherapy had a better quality of life (*p* = 0.03). The studies showed a null level of heterogeneity (I^2^ = 0%) (Fig. 2A). When analyzing pain, a reduction was also observed in the groups treated with electrotherapy or electrotherapy plus pelvic floor muscle training, with an SMD of -4.43 (95% CI: -7.9 to -0.96) (*p* = 0.01). The studies, in this case, showed a high level of heterogeneity (I^2^ = 98%) (Fig. 2b). When analyzing sexual function, there was no significant difference between the groups analyzed (*p* = 0.22), with an SMD of 2.37 (95% CI: -1.43 to 6.17). These studies showed a high level of heterogeneity (I^2^ = 97%) (Fig. 2c).

### Risk of bias for individual studies

The risk of bias within individual studies was determined to be critical in six studies (31.6%) [19, 24, 26–28, 32] while six studies had a low risk of bias [20, 25, 30, 31, 34, 35]. The remaining studies were classified as having a moderate risk of bias [17, 18, 23, 29, 33] (Table 4).

**Table 4:** Risk of bias for included studies

ROBINS-I tool results for non-randomized studies								
**Authors**	**Confounding**^**a**^	**Selection**^**b**^	**Classification of interventions**	**Derivation from intended intervention**	**Missing data**^**c**^	**Outcomes**	**Selective reporting**^**d**^	**Overall**
Alshiek et al. (2017) [26]	Critical	Critical	Low	Low	Low	Low	Critical	Critical
Brotto et al. (2015) [32]	Critical	Critical	Low	Low	Low	Low	Critical	Critical
Cyr et al. (2020) [19]	Critical	Critical	Low	Low	Low	Low	Critical	Critical
Cyr et al. (2021) [22]								
Cyr et al. (2022) [21]								
Da Silva et al. (2016) [29]	Critical	Low	Low	Low	Low	Low	Critical	Moderate
Del Forno et al. (2020) [28]	Critical	Critical	Low	Low	Low	Low	Critical	Critical
Fernández-Cuadros et al. (2020) [27]	Critical	Critical	Low	Low	Low	Low	Critical	Critical
Yong et al. (2018) [31]	Low	Low	Low	Low	Low	Low	Critical	Low
**RoB tool results for randomized studies**								
**Authors**	**Random sequence (selection bias)**	**Allocation concealment (selection bias)**	**Blinding of participants and personnel (performance bias)**	**Blinding of outcome assessment (detection bias)**	**Incomplete outcome data (attrition bias)**	**Selective reporting (reporting bias)**	**Other bias**	**Overall**
Del Forno et al. (2021) [30]	Low	Low	High	Low	Low	Low	Low	Low
Franco et al. (2021) [34]	Low	Low	High	Low	Low	Low	Low	Low
Ghaderi et al. (2019) [17]	Low	High	High	Low	Low	Low	Low	Moderate
Hurt et al. (2021) [35]	Low	Low	HIgh	Low	Low	Low	Low	Low
Kolberg et al. (2015) [33]	Low	High	High	Low	Low	Low	Low	Moderate
Mira et al. (2015) [23]	Low	High	High	High	Low	Low	Low	Moderate
Mira et al. (2020) [24]	Low	High	High	High	High	Low	Low	Low
Morin et al. (2021) [20]	Low	Low	High	Low	Low	Low	Low	Low
Murina et al. (2018) [25]	Low	High	Low	Low	Low	Low	Low	Low
Schvertzman et al. (2019) [18]	Low	High	High	High	Low	Low	Low	Moderate

^a^ Risk of bias from confounding was considered critical when confounding was not inherently controlled for (i.e. no or limited adjustment)

^b^ Selection bias was critical when selection into the study was very strongly related to intervention and outcome. This occurred when the study included women with diagnoses other than dyspareunia

^c^ Risk of bias due to missing data was considered moderate when there appeared to be a substantial amount of missing data. In these cases, the proportions of and reasons for missing data might differ across interventions groups. Of note, the majority of studies did not report on missing data. The risk of bias for these were classified as low, but could also be considered “unknown”

^d^ The studies with a moderate risk for selective outcome reporting were those that did not provided a pre-registered protocol

Additionally, the certainty of the evidence obtained was assessed as low for the variables of pain and quality of life, and very low for the variable of sexual function (Table 5).

**Table 5:** Certainty of the evidence (GRADE)

Outcomes	Number of participants (studies)	Risk of bias^a^	Inconsistency^b^	Indirectness	Imprecision	Other considerations	Certainty of the evidence (GRADE)
Pain	456 (4 RCTs)	Low	Serious	Not serious	Low	None	⨁⨁◯◯ Moderate
Sexual function	373 (2 RCT)	Not seious	Serious	Not serious	Serious	None	⨁◯◯◯Low
Quality of life	64 (2 RCTs)	Serious	Not serious	Not serious	Low	None	⨁◯◯◯Low

*RCT* randomized clinical trial, *SMD* standardized mean difference

^a^ The average risk of bias of the studies according to the ROBINS-I and RoB tools was good

^b^ Low methodological heterogeneity but high statistical heterogeneity among trials (I^2^ > 25%)

## Discussion

The objective of this study was to assess the effectiveness of physical therapy interventions in treating female dyspareunia. The applied techniques were found to be effective, and significant differences were observed among the different modalities of physiotherapy that were studied.

All the reviewed articles consistently reported a significant reduction in pain intensity [17, 19, 20, 23–26, 28–30, 32, 33, 35]. The studies that employed multimodal physical therapy interventions, including techniques such as TENS, manual therapy, pelvic floor muscle training, and education, showed superior improvements in pain intensity compared to other interventions [17]. Although it is not the only study that used this type of intervention [18–22], it is the only one that incorporated high-frequency TENS, the purpose of which is strictly antalgic. This type of electrical stimulation activates afferent fibers and inhibits the response of nociceptive fibers through the activation of interneurons in the gray matter of the spinal cord's posterior horn [37]. The meta-analysis conducted further supports the effectiveness of electrotherapy in reducing pain and, consequently, improving quality of life.

The improvements observed in pain intensity indirectly suggest improvements in sexual function [38]. Several studies specifically measured sexual function and reported positive results [17–20, 24, 29, 32] (except for when pelvic floor muscle training was applied in isolation [34]). However, these improvements did not reach statistical significance in the meta-analysis.

Of note is the study by Ghaderi et al. [17], which showed the most significant difference in the Female Sexual Function Index score immediately after treatment, and these improvements were sustained three months later. Except for two studies [24, 29], the remaining studies included pelvic floor muscle training as part of the intervention [17–20, 32, 34]: either as home exercises [32], in-clinic sessions [18] or a combination of clinic-based training with a daily home exercise program [17, 19, 20, 34].

The combination of pelvic floor muscle training with other treatment strategies in a multimodal intervention has been shown to yield the best results for improving sexual function [17, 19, 20]. It is important to note that sexual function is a complex construct influenced by various factors, which explains why pelvic floor muscle training alone did not lead to significant improvements in the study by Franco et al. [34].

Training the perineal musculature has multiple benefits, including improving relaxation capacity, restoring normal resting activity, increasing vaginal elasticity, and enhancing muscle awareness and proprioception. These effects help reverse connective tissue and myofascial damage associated with pelvic-perineal pain and dysfunction [39, 40]. Furthermore, a previous review has suggested that weakness in this musculature contributes to a woman's inability to reach orgasm, which is often observed in cases of dyspareunia [41]. Additionally, having good tone in the muscles attached to the clitoral corpus cavernosum, such as the ischiocavernosus and bulbo-spongiosus muscles, can enhance the involuntary contraction of the perineal musculature, thereby improving arousal and orgasmic response [42, 43].

Indeed, the success of an active kinesitherapy program for pelvic floor muscle training relies on several key factors. First, it is important to have professional supervision to ensure proper technique and progress in the exercises. Additionally, combining the training program with other complementary techniques can enhance its effectiveness. Providing clear instructions for performing exercises at home is crucial for maintaining continuity and achieving optimal results [41]. The principles of specificity, overload, reversibility, and duration should be considered when designing an exercise program for pelvic floor muscles [43]. This means that the training should be tailored to the individual needs of the patient, with progressive increases in load and difficulty as the muscle response improves. The program should be continued for as long as necessary to bring about functional changes. Rather than solely focusing on strength gain, emphasis should be placed on coordination and relaxation capacity. It is also important to address breathing normalization and correct posture as part of the training program [39]. It should be noted that not all of these guidelines were fully implemented in the interventions analyzed, except in the study by Ghaderi et al. [17]. Their program included supervised and progressive exercises, combined with manual therapy techniques, electrotherapy, and patient education. They also provided additional information and a diary for recording compliance with the exercises, following recommendations from relevant publications [39, 43]. This comprehensive approach likely contributed to their successful outcomes. Thiele massage is indeed a technique that has shown positive short-term effects on sexual functionality [28, 29]. Da Silva et al. [29] applied this technique for four weekly sessions, each lasting five minutes, and achieved greater improvements compared to other studies that used more complex and prolonged treatments [18–20, 24, 32, 34].

The severity of dyspareunia was also reduced with physiotherapy interventions [20, 24, 31, 32, 35], congruent with the results obtained. However, the best results were achieved with the application of multimodal physiotherapy treatment [20] and the use of extracorporeal shockwave therapy [35]. The particularity of the multimodal treatment lies in the application of trigger point myofascial release techniques. For this treatment, manual therapy techniques aimed at improving blood flow, vulvar and visceral mobility, relieving nerve compression and pain, include myofascial release [39]. Myofascial release is used to bring the muscle to its optimal length, decrease pain, and improve function [44]. Therefore, the treatment of trigger points, which represent one of the etiological factors of dyspareunia, should be considered as one of the techniques in approaching this condition. Simultaneously, the positive results identified with the use of extracorporeal shockwave therapy are consistent with its ability to improve chronic regional inflammation in the vulvar and vaginal area. The physical forces generated by low-intensity shockwaves affect tissue mechanics and can trigger the release of growth factors and anti-inflammatory factors [45, 46].

Regarding muscular properties, all the studies that evaluated them showed improvement with physiotherapy interventions [17, 18, 21, 25, 27, 28, 30, 33].

The studies that included pelvic floor muscle training in combination with biofeedback achieved this improvement with a lower number of sessions [18, 21, 27]. This method facilitates the learning of contraction and relaxation commands and, as a result, reinforces the training performed [47, 48]. An additional benefit is that this technique has no contraindications, making it recommended to help patients optimize the effects of training, improve their self-confidence, and increase their perception and knowledge of their own body [47].

This research has several limitations. Firstly, the sample heterogeneity is a limitation, as some studies included patients with dyspareunia associated with other diseases [19, 21–25, 28, 29, 32], or did not solely focus on dyspareunia patients [27, 33]. Furthermore, only two studies took into account whether the dyspareunia was superficial or deep, and the diagnostic methods for dyspareunia varied widely among the studies. The interventions applied also showed a high degree of heterogeneity, with different techniques, durations, and frequencies of sessions being used. Additionally, most of the publications had low risk of bias [19–22, 24–28, 30–32, 34, 35]. As a result of these characteristics, the certainty of the evidence obtained is low. However, we must recognize the strengths of this work, which include being the most complete and extensive review with meta-analysis to date. It has taken into account all the therapeutic options for dyspareunia available in physiotherapy as well as their different modalities of intervention.

Further research is needed to approach dyspareunia from a physiotherapy perspective and establish a standardized treatment protocol. More randomized controlled trials comparing different treatment strategies, dosages, and durations of sessions are necessary. Additionally, other variables related to dyspareunia, such as the personal relationship with the partner and the female sexual response, should be investigated. It is also important to explore sociocultural and psychological factors associated with dyspareunia.

## Conclusion

Physiotherapy techniques have shown to be effective in improving pain and quality of life in women with dyspareunia. One crucial aspect is the strengthening of the perineal muscles, which should be carried out both in clinical settings and at home with proper guidance and informative materials. Combining perineal muscle strengthening with biofeedback training, which enhances somatosensory sensitization, and the application of TENS at a high frequency and intensity below the pain threshold has demonstrated positive effects on these patients. Additionally, manual trigger point release therapy and Thiele massage have been proven to effectively reduce pain intensity.

It is important to note that dyspareunia is not solely a physical disorder, but it also has significant psychological implications. Therefore, the involvement of mental health professionals in the treatment process is crucial to enhance the overall quality of life for participants. By combining these techniques with educational guidelines for women, the likelihood of successful treatment outcomes can be increased.

## Data Availability

The datasets used and/or analyzed during the current study are available from the corresponding author on reasonable request.

## References

[1] Clayton AH, Juarez EMV (2019). Female sexual dysfunction. Med Clin North Am.

[2] McCool-Myers M, Theurich M, Zuelke A (2018). Predictors of female sexual dysfunction: a systematic review and qualitative analysis through gender inequality paradigms. BMC Womens Health.

[3] Raina R, Pahlajani G, Khan S (2007). Female sexual dysfunction: classification, pathophysiology, and management. Fertil Steril.

[4] Shifren JL, Monz BU, Russo PA, Segreti A, Johannes CB (2008). Sexual problems and distress in United States women: prevalence and correlates. Obstet Gynecol.

[5] Alimi Y, Iwanaga J, Oskouian RJ (2018). The clinical anatomy of dyspareunia: a review. Clin Anat.

[6] Harris V, Fischer G, Bradford JA (2017). The aetiology of chronic vulval pain and entry dyspareunia: a retrospective review of 525 cases. Aust N Z J Obstet Gynaecol.

[7] Orr N, Wahl K, Joannou A (2020). Deep dyspareunia: review of pathophysiology and proposed future research priorities. Sex Med Rev.

[8] Weinberger JM, Houman J, Caron AT (2019). Female sexual dysfunction: a systematic review of outcomes across various treatment modalities. Sex Med Rev.

[9] Areskoug-Josefsson K, Gard G (2015). Physiotherapy as a promoter of sexual health. Physiother Theory Pract.

[10] Cumpston M, Li T, Page MJ (2019). Updated guidance for trusted systematic reviews: a new edition of the cochrane handbook for systematic reviews of interventions. Cochrane Database Syst Rev.

[11] Ardern CL, Büttner F, Andrade R (2022). Implementing the 27 PRISMA 2020 statement items for systematic reviews in the sport and exercise medicine, musculoskeletal rehabilitation and sports science fields: The PERSiST (implementing prisma in exercise, rehabilitation, sport medicine and SporTs science) guidance. Br J Sports Med.

[12] Sterne JA, Hernan MA, Reeves BC (2016). ROBINS-I: a tool for assessing risk of bias in non-randomised studies of interventions. BMJ.

[13] Cochrane Methods Bias. RoB 2: A revised Cochrane risk-of-bias tool for randomized trials; 2023. Available from: https://methods.cochrane.org/bias/resources/rob-2-revised-cochrane-risk-bias-tool-randomized-trials

[14] Guyatt GH, Oxman AD, Vist GE (2008). GRADE: an emerging consensus on rating quality of evidence and strength of recommendations. BMJ.

[15] Higgins JPT, Green S, The Cochrane Collaboration (2011). Cochrane handbook for systematic reviews of interventions. Version 5.1.0..

[16] Higgins JPT, Altman DG, Gøtzsche PC (2011). Cochrane statistical methods group. The cochrane collaboration’s tool for assessing risk of bias in randomised trials. BMJ.

[17] Ghaderi F, Bastani P, Hajebrahimi S (2019). Pelvic floor rehabilitation in the treatment of women with dyspareunia: a randomized controlled clinical trial. Int Urogynecol J.

[18] Schvartzman R, Schvartzman L, Ferreira CF (2019). Physical therapy intervention for women with dyspareunia: a randomized clinical trial. J Sex Marital Ther.

[19] Cyr M, Dumoulin C, Bessette P (2020). Feasibility, acceptability and effects of multimodal pelvic floor physical therapy for gynecological cancer survivors suffering from painful sexual intercourse: a multicenter prospective interventional study. Gynecol Oncol.

[20] Morin M, Dumolin C, Bergeron S (2021). Multimodal physical therapy versus topical lidocaine for provoked vestibulodynia: a multicenter, randomized trial. Am J Obstet Gynecol.

[21] Cyr MP, Dumolin C, Bessette P (2022). Changes in pelvic floor morphometry and muscle function after multimodal physiotherapy for gynaecological cancer survivors sufferinf from dyspareunia: a prospective interventional study. Physiotherapy.

[22] Cyr MP, Dumolin C, Bessette P (2021). A prospective single-arm study evaluating the effects of a multimodal physical therapy intervention on psychosexual outcomes in women with dyspareunia after gynecologic cancer. J Sex Med.

[23] Mira TA, Giraldo PC, Yela DA (2015). Effectiveness of complementary pain treatment for women with deep endometriosis through transcutaneous electrical nerve stimulation (TENS): randomized controlled trial. Eur J Obstet Gynecol Reprod Biol.

[24] Mira TA, Yela DA, Podgaec S (2020). Hormonal treatment isolated versus hormonal treatment associated with electrotherapy for pelvic pain control in deep endometriosis: randomized clinical trial. Eur J Obstet Gynecol Reprod Biol.

[25] Murina F, Felice R, Di Francesco S (2018). Vaginal diazepam plus transcutaneous electrical nerve stimulation to treat vestibulodynia: a randomized controlled trial. Eur J Obstet Gynecol Reprod Biol.

[26] Alshiek J, Garcia B, Minassian V (2020). Vaginal energy-based devices. Female Pelvic Med Reconstr Surg.

[27] Fernández-Cuadros ME, Kazlauskas SG, Albaladejo-Florin MJ (2020). Effectiveness of multimodal rehabilitation (biofeedback plus capacitive-resistive radiofrequency) on chronic pelvic pain and dyspareunia: Prospective study and literature review. Rehabilitacion.

[28] Del Forno S, Arena A, Alessandrini M (2020). Transperineal ultrasound visual feedback assisted pelvic floor muscle physiotherapy in women with deep infiltrating endometriosis and dyspareunia: a pilot study. J Sex Marital Ther.

[29] Da Silva APM, Montenegro ML, Gurian MBF (2017). Perineal massage improves the dyspareunia caused by tenderness of the pelvic floor muscles. Rev Bras Ginecol Obstet.

[30] Del Forno S, Arena A, Pellizzone V (2021). Assessment of levator hiatal area using 3D/4D transperineal ultrasound in women with deep infiltrating endometriosis and superficial dyspareunia treated with pelvic floor muscle physiotherapy: randomized controlled trial. Ultrasound Obstet Gynecol.

[31] Yong PJ, Williams C, Bodmer-Roy S (2018). Prospective cohort of deep dyspareunia in an interdisciplinary setting. J Sex Med.

[32] Brotto LA, Yong P, Smith KB (2015). Impact of a multidisciplinary vulvodynia program on sexual functioning and dyspareunia. J Sex Med.

[33] Kolberg M, Hilde G, Stær-Jensen J (2016). Effect of postpartum pelvic floor muscle training on vaginal symptoms and sexual dysfunction—secondary analysis of a randomised trial. BJOG.

[34] Franco MM, Pena CC, de Freitas LM (2021). Pelvic floor muscle training effect in sexual function in postmenopausal women: A randomized controlled trial. J Sex Med.

[35] Hurt K, Zahalka F, Halaska M, Rakovicova I, Rakovic J, Cmelisnky V (2021). Extracorporeal shock wave therapy for treating dyspareunia: A prospective, randomized, double-blind, placebo-controlled study. Ann Phys Rehabil Med.

[36] Trahan J, Leger E, Allen M (2019). The efficacy of manual therapy for treatment of dyspareunia in females: a systematic review. J Womens Health Phys Ther.

[37] Sharma N, Rekha K, Srinivasan JK (2017). Efficacy of transcutaneous electrical nerve stimulation in the treatment of chronic pelvic pain. J Midlife Health.

[38] Meana M, Binik YM, Khalifé S (1997). Biopsychosocial profile of women with dyspareunia. Obstet Gynecol.

[39] Murphy KM, Fosnight A (2018). The role of pelvic floor physical therapy for the female patient. Physician Assist Clin.

[40] Wallace SL, Miller LD, Mishra K (2019). Pelvic floor physical therapy in the treatment of pelvic floor dysfunction in women. Curr Opin Obstet Gynecol.

[41] Ferreira CHJ, Dwyer PL, Davidson M (2015). Does pelvic floor muscle training improve female sexual function? A systematic review. Int Urogynecol J.

[42] Jannini EA, Buisson O, Rubio-Casillas A (2014). Beyond the G-spot: Clitourethrovaginal complex anatomy in female orgasm. Nat Rev Urol.

[43] Johnson VY (2001). How the principles of exercise physiology influence pelvic floor muscle training. J Wound Ostomy Continence Nurs.

[44] Ajimsha MS, Al-Mudahka NR, Al-Madzhar JA (2015). Effectiveness of myofascial release: systematic review of randomized controlled trials. J Bodyw Mov Ther.

[45] Lowenstein L, Vardi Y, Deutsch M (2004). Vulvar vestibulitis severity - Assessment by sensory and pain testing modalities. Pain.

[46] Mariotto S, de Prati AC, Cavalieri E (2009). Extracorporeal shock wave therapy in inflammatory diseases: molecular mechanism that triggers anti-inflammatory action. Curr Med Chem.

[47] Hite M, Curran T (2021). Biofeedback for pelvic floor disorders. Clin Colon Rectal Surg.

[48] Aalaie B, Tavana B, Rezasoltani Z, Aalaei S, Ghaderi J, Dadarkhah A (2021). Biofeedback versus electrical stimulation for sexual dysfunction: a randomized clinical trial. Int Urogynecol J.

